# The Lipid Bilayer Provides a Site for Cortisone Crystallization at High Cortisone Concentrations

**DOI:** 10.1038/srep22425

**Published:** 2016-03-03

**Authors:** Richard J. Alsop, Adree Khondker, Jochen S. Hub, Maikel C. Rheinstädter

**Affiliations:** 1 Department of Physics and Astronomy, McMaster University, Hamilton, Ontario, Canada; 2 Institute for Microbiology and Genetics, Georg-August-University Göttingen, Göttingen, Germany

## Abstract

Cortisone is an injected anti-inflammatory drug that can cause painful side effects known as “steroid flares” which are caused by cortisone crystallizing at the injection site. We used molecular dynamics simulations and X-ray diffraction to study the interaction of cortisone with model lipid membranes made of 1-palmitoyl-2-oleoyl-sn-glycero-3-phosphocholine (POPC) at drug concentrations from 0 mol% to 50 mol%. Cortisone was found to partition in the lipid bilayer and locate in the hydrophilic to hydrophobic interface of the membranes. Cortisone strongly affects the integrity of the membrane, as quantified by a decreased membrane thickness, increased area per lipid, and decreased lipid tail order parameters. At cortisone concentrations of more than 20 mol%, signals from crystallized cortisone were observed. These crystallites are embedded in the bilayers and orient with the membranes. While the cortisone molecules align parallel to the bilayers at low concentrations, they start to penetrate the hydrophobic core at higher concentrations. Trans-membrane crystallites start to nucleate when the membrane thickness has decreased such that cortisone molecules in the different leaflets can find partners from the opposite leaflet resulting in a non-zero density of cortisone molecules in the bilayer center. We suggest that the lipid bilayer provides a site for cortisone crystallization.

In the pharmaceutical industry, drugs are typically developed to interact directly with molecular targets, such as protein receptors, and these targets (or receptors) may be embedded in cell membranes^1^,^2^. However, a drug molecule, such as cortisone, can also directly influence the lipid membrane via physical interactions, thereby changing the properties and functioning of the membrane^3^,^4^,^5^. These non-specific interactions are believed to often be responsible for drug side effects, as changes to the membrane’s properties can influence the function of membrane-embedded proteins^4^,^5^.

Cortisone (17-hydroxy-11-dehydrocorticosterone) is a synthetic glucocorticoid used globally and listed under the World Health Organization’s Essential Medicines^6^. Glucocorticoids are a subcategory of steroids, which have anti-inflammatory properties^7^,^8^.

Cortisone can be taken orally to treat general inflammation, although it is more commonly used to treat local joint inflammation by direct injection to the active site. Cortisone primarily acts by specifically binding to an intracytoplasmic nuclear receptor, forming a complex, which enters the nuclear membrane and interacts with basic transcription factors^9^,^10^. This causes the release of lipocortins, thereby inhibiting the production of prostaglandins, and leukotrienes, which reduce inflammation^11^,^12^,^13^,^14^.

However, there are several severe side-effects of cortisone, which include muscle wasting, hyperglycemia, and steroid psychosis^15^,^16^,^17^. These side-effects have partially been explained by non-specific interactions with lipid membranes^18^. In addition, corticosteroid injections can cause so-called “steroid flares”, which are characterized by pain at the site of injection and typically occur within 1–2 days of injection^19^,^20^. Insoluble, *μ*m sized cortisone crystals forming on the synovial membrane cause macrophages to collect at the cite of crystallization. The immune response, known as a flare, leads to the release of synovial fluid, swelling, and pain at the site of injection^21^,^22^,^23^.

X-ray diffraction experiments have shown cortisol, the antiderivative of cortisone, which differs from cortisone only by a hydroxyl group in place of a ketone group, to localize within the bilayer at *z*-values of 10 Å < *z* < 19 Å^24^ (*z* = 0 marks the bilayer center). In addition, MD simulations of cortisone show that it is able to localize in the head group-tail group interface of bilayer; however, dependent upon initial position^25^. The simulations suggest the polar groups on the cortisone molecule interact with the choline, glycerol, and phosphate groups of the lipid molecules.

By combining MD simulations and high resolution X-ray diffraction we show that cortisone crystallizes inside of lipid membranes at high cortisone concentrations. Unsaturated 1-palmitoyl-2-oleoyl-sn-glycero-3-phosphocholine (POPC) membranes were studied at cortisone concentrations between 0 and 50 mol%. Cortisone was found to partition in the lipid bilayers and to locate between the hydrophilic and hydrophobic regions, orienting parallel to the membranes. The presence of cortisone leads to a continuous thinning of the bilayers and an increase of the lipid area at concentrations of up to 20 mol%. At higher concentrations, cortisone was found to strongly penetrate the hydrophobic core and eventually nucleate in 2-dimensional crystallites inside the membranes. This mechanism may be related to the formation of steroid flares in cortisone therapy in biological tissue.

## Results

### Molecular Dynamics Simulations

**Figure 1:**
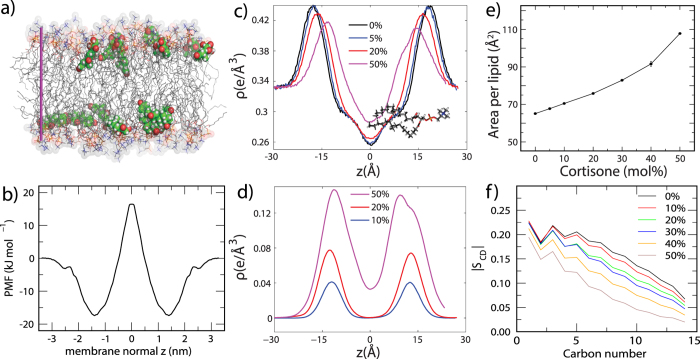
(**a**) A simulation snapshot of a POPC membrane containing 10 mol% cortisone; POPC is show as sticks, cortisone as solid spheres. Water is not shown for clarity. (**b**) Potential of mean force (PMF) for cortisone across along the membrane normal *z. z* = 0 corresponds to the membrane center. (**c**) Electron density profiles calculated from simulations. (**d**) electron density of cortisone. (**e**) Area per phospholipid molecule as a function of cortisone content. (**f**) Deuterium order parameter of the saturated POPC tail as calculated from the simulations.

We used MD simulations to identify the energetics and conformations of cortisone in the membranes, as well as the effects of cortisone on the structure of the lipid bilayer. A typical simulation snapshot for a concentration of 10 mol% cortisone is shown in Fig. 1a.

First, we computed the potential of mean force (PMF) for a single cortisone molecule across the pure POPC membrane (Fig. 1b). The PMF suggests an average membrane/water partition coefficient of 220, demonstrating a strong preference of cortisone for the membrane over bulk water. In line with a previous simulation study^25^, the PMF exhibits pronounced minima at a distance of 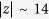 Å from the bilayer center. Thus, at low cortisone concentrations, the molecule preferentially binds at the head group-tail interfaces of the membrane. Visual inspection of the simulations showed that, at this position, cortisone predominantly binds with the hydrophobic edge of the sterol groups oriented towards the hydrophobic tails, thereby minimizing any hydrophobic/hydrophilic mismatch.

In order to compare the simulations to our X-ray scattering data (see below), we further computed electron density profiles from equilibrium simulations at various cortisone concentrations. Figure 1c presents the overall density profiles, representing POPC, cortisone and water. The electron density at the edge of the bilayers is found to be 0.33 e^−^/Å^3^ corresponding to bulk water. The maximum in density at 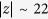 Å is caused by the electron rich phospholipid head group region. Upon the addition of cortisone, the density peaks continuously shifts towards the center of the membranes, indicative of a decrease in bilayer thickness.

Further insight into cortisone conformations is given by the density purely from cortisone molecules in Fig. 1d. At low cortisone content, all cortisone molecules are localized at the head-group tail-group interface (near the glycerol moiety) as suggested by the single-cortisone PMF. At high local cortisone concentration, however, the molecules begin to span the entire membrane, as indicated by a non-zero cortisone density at 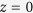.

To quantify the effect of cortisone on the structure of membrane, we computed the area per phospholipid 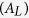 as well as the the deuterium order parameter 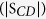 of the saturated POPC tail as a function of cortisone content, as shown in Fig. 1e,f. We observed a large increase in 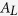 from 65 Å^2^ at 0% cortisone up 105 Å^2^ at 50% cortisone. The lipid area for the pure POPC bilayer is in good agreement with the experimental value of 68.3 Å^2 ^26. Simultaneously, the order of the lipid tails strongly decreases with increasing cortisone content. Taken together, the simulations highlight that the membrane does not merely provide a passive solvent for the accumulation of cortisone. Instead, cortisone has drastic effects on the integrity of the membrane, leading to a decreased bilayer thickness, increased area per lipid, and increased tail disorder.

### X-ray Diffraction of POPC Membranes with Cortisone

Highly oriented, multi-lamellar membrane stacks were prepared on silicon wafers and the molecular structure was studied using high resolution X-ray diffraction imaging. By using oriented membranes, the in-plane structure 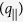 and the out-of-plane structure 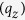 were determined independently, however, simultaneously. All membranes were scanned at T = 28 °C and 97% relative humidity (RH), in the fluid state of the POPC bilayers.

**Figure 2:**
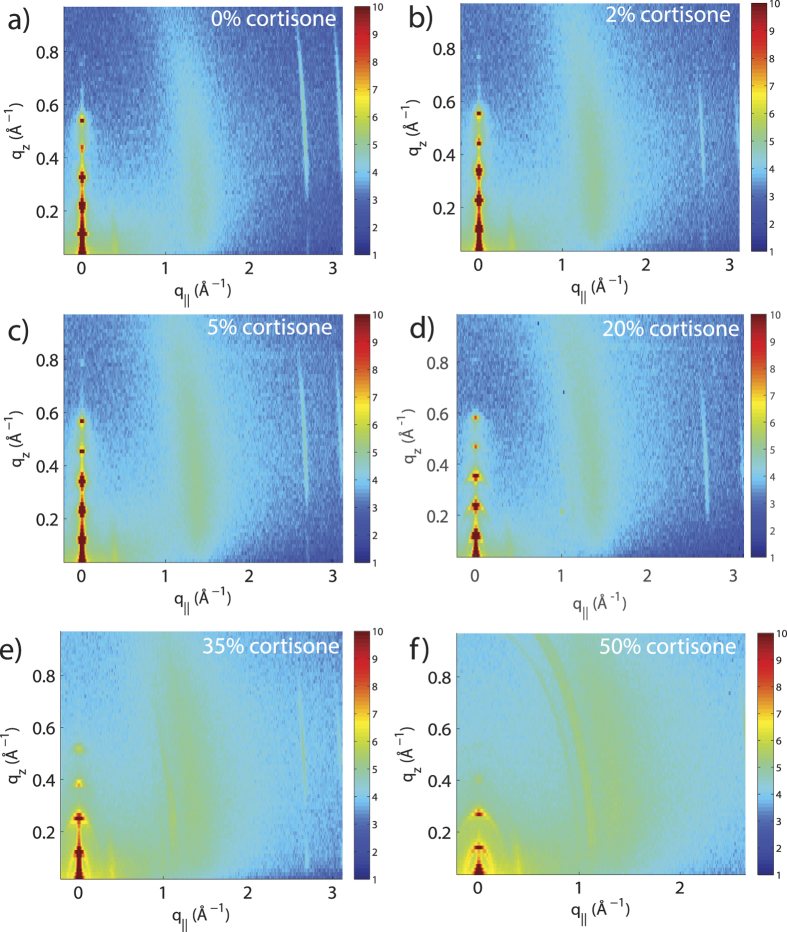
Two-dimensional X-ray intensity maps of bilayers with increasing cortisone concentration. (**a**) 0 mol%, (**b**) 2 mol%, (**c**) 5 mol%, (**d**) 20 mol%, (**e**) 35 mol% and (**f**) 50 mol% cortisone. All samples exhibit well-spaced Bragg peaks along 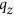, and a broad peak at 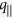, indicative of oriented fluid-phase membranes. At >20 mol%, additional peaks are observed in-plane indicating crystallization of cortisone.

Two-dimensional X-ray intensity maps for cortisone concentrations of 0, 2, 5, 10, 35 and 50 mol% cortisone are shown in Fig. 2. The pure POPC sample in Fig. 2a shows five well-developed Bragg peaks along the 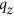 axis, indicative of well organized lamellar bilayers. A single, broad in-plane peak at 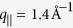 is the result of the hexagonal packing of the lipid tails in the membrane core and the tell-tale of a fluid, disordered structure^27^. In line with scattering intensities computed from the simulations (Figure S1), this tail correlation peak is smeared out with increasing cortisone content, indicative for increasing disorder of the lipid tails. Additional in-plane reflections are observed for cortisone concentrations of 20 and 35 mol%, indicative of the formation of cortisone crystallites. 20 mol% was, therefore, defined as the experimental solubility limit of cortisone in POPC bilayers.

**Figure 3:**
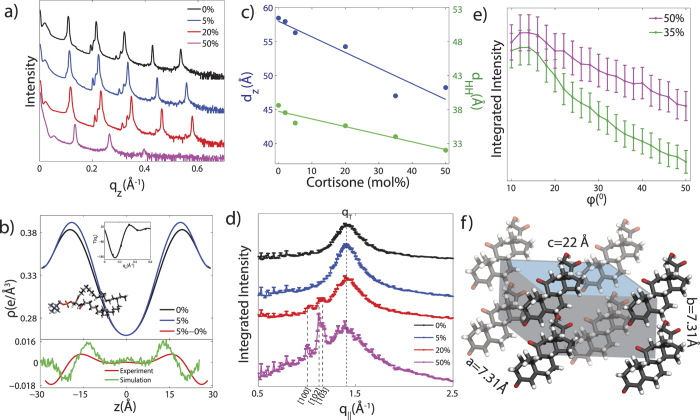
(**a**) Out-of plane X-ray diffraction of oriented POPC membranes containing cortisone. (**b**) Top: scaled electron densities across the axis perpendicular to the bilayer for 0 mol% and 5 mol% cortisone. Bottom: the difference of these curves shows a positive peak at 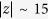 Å, indicating the position of cortisone, and a negative peak at 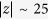 Å due to the thinning of the membrane. The difference curve from the MD simulations at 5 mol% is included for comparison. The slight disagreement in peak position and width between experiment in simulation is likely due to bilayer undulations^28^. An example of 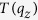 is shown as an inset. (**c**) The lamellar spacing, 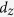, and the head-head distance, 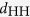, as a function of cortisone concentration as determined from diffraction experiments. (**d**) Scattering along 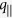 for the oriented membrane samples. Additional peaks, which appear for cortisone concentrations >20 mol%, can be indexed by crystalline cortisone. (**e**) The integrated intensities for the crystalline cortisone peak observed at 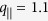 Å^−1^ as a function of azimuthal angle *ϕ*. (**f**) Unit cell of the observed cortisone crystallites, as determined from the in-plane Bragg peaks.

Electron density profiles 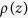 of the bilayers were determined through Fourier analysis of the out-of-plane Bragg peaks shown in Fig. 3a. 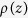 for the pure POPC bilayer and POPC + 5 mol% cortisone are shown in Fig. 3b (top). In addition, Fig. 3b (bottom) presents change in electron density upon the addition of 5% cortisone, computed from the Bragg peaks (red curve) and from the MD simulations (green curve). We find that, upon the addition of cortisone, the electron density increases at 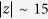 Å, which corresponds to the position of cortisone molecules, and the density decreases at 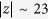 Å due to thinning of the membrane. The difference between experimental and simulation peak positions (2–3 Å) and width likely arise due to bilayer undulations present in the bilayer stack^28^. The experiments thus locate cortisone inside the bilayers, between the head group and tail group region, in agreement with the MD simulations.

The lamellar spacing, 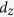, and head group to head group spacing, 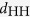, are depicted in Fig. 3c. Both spacings significantly decrease with increasing cortisone concentrations. The width of the head group region can be estimated from the width of the corresponding peak in the electron densities from MD simulations and experiment to be ~7 Å. The lamellar spacing at a cortisone concentration of 50 mol% is determined to be *d*_*z*_ = 48 Å, the head group distance to 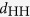 = 32 Å and the thickness of the hydrophobic core to ~25 Å. The fact that the lamellar spacing decreased slightly faster than the membrane thickness with increasing cortisone content is strong evidence that the cortisone molecules partition in the bilayers and cortisone crystallites do not form outside of the membranes.

In-plane diffraction is shown in Fig. 3d. While 0, 2 and 5 mol% cortisone membranes show the lipid acyl chain correlation peak, only, additional peaks are observed at 20 mol% and 50 mol% whose intensities increase with increasing cortisone. The peaks at 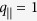 Å^−1^, 1.12 Å^−1^, and 1.17 Å^−1^ agree well with the [100], [102], [103] reflections observed from crystallized cortisone^29^, respectively, with lattice constants of *a* = 7.31 Å and *c* = 22 Å. The presence of these peaks suggests the formation of crystallized cortisone at high concentrations.

To determine the orientation of the cortisone structures with respect to the bilayers, the peaks at 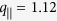 Å^−1^ and 1.17 Å^−1^ were integrated along the azimuthal angle *ϕ* from 10° < *ϕ* <60° ( *ϕ* <10 was not included due to the effect of absorption). The results for 35 mol% and 50 mol% cortisone are presented in Fig. 3e. Herman’s orientation function, 
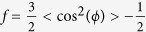
, was used to quantify the orientation of the crystals with the membrane stack. Crystals observed in the 35 mol% sample result in *f* = 0.96 and crystals from the 50 mol% sample show *f* = 0.64. While a perfect orientation has *f* = 1, randomly oriented crystallites would result in *f* = 0.25. The cortisone crystals, therefore, have a preferred orientation along 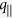. The organization of the cortisone molecules and the corresponding unit cell is depicted in Fig. 3f.

## Discussion

The interaction of cortisone with model POPC bilayers was studied using MD simulations and X-ray diffraction. In both, experiment and simulation, cortisone was found to strongly interact with the lipid bilayer. X-ray and neutron diffraction experiments have previously reported conformational changes in lipid bilayers with the introduction of drugs and hormones^30^,^31^,^32^,^33^. Amphiphilic drugs, such as aspirin or ibuprofen were shown to partition into the interfacial region^31^,^32^,^33^,^34^.

A membrane bound state for cortisone was found in MD simulations and X-ray diffraction experiments at low cortisone concentrations. The simulations observed a minima in the PMF for cortisone across the bilayer, with a strong preference to avoid entering the bilayer center. Simulations and experiments observe cortisone in the hydrophilic to hydrophobic interface with the hydrophobic face in contact with the lipid tails. By positioning into the interfacial region of the bilayer, cortisone causes a decrease in the bilayer thickness and an increase in the area per lipid molecule.

Additional scattering features were observed at 20 mol% cortisone, indicating the presence of crystallized cortisone within the membranes. These cortisone crystals are oriented with the membrane; the corresponding crystal lattices are forming perpendicular to the membrane plane. During the simulations, however, no evidence for cortisone crystallization was observed, neither from visual inspection of the trajectories nor from computed X-ray intensity maps (Figure S1), suggesting that crystallization occurs on time scales beyond the accessible simulation times. The lipids provide a viscous environment for cortisone, which leads to slow conformational sampling of cortisone-cortisone contacts, which would be required for crystallization. Alternatively, we cannot exclude the possibility that the applied force fields do not reproduce the free-energy difference between crystallized and solubilized cortisone.

**Figure 4:**
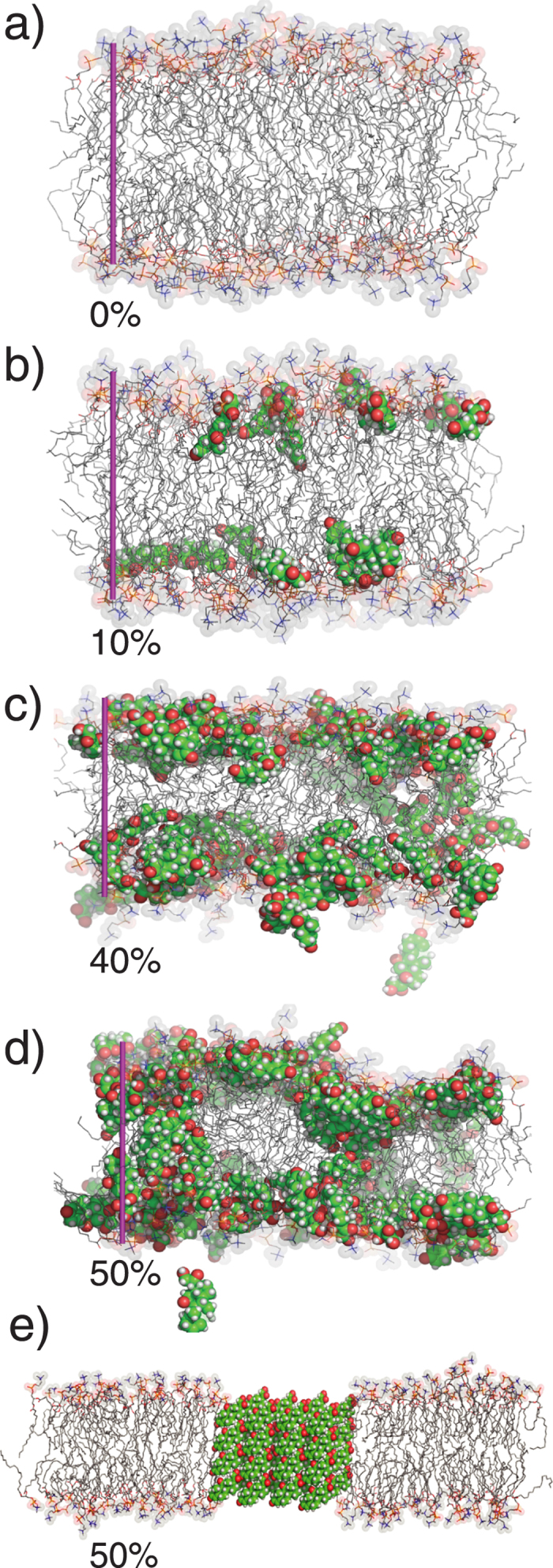
Simulation snapshots for POPC membranes at 0, 10, 40 and 50 mol% (**a**–**d**). The purple bar indicates a 40 Å ruler. The structural model of the cortisone crystals based on X-ray experiments is shown in (**e**).

The process of cortisone crystallite formation is illustrated in Fig. 4. Parts a) to d) show simulation snapshots for cortisone concentrations of 0, 10, 40 and 50 mol%. At low cortisone concentrations of 10 mol%, the cortisone molecules partition in the hydrophilic/hydrophobic bilayer interface and align parallel to the bilayers. The presence of cortisone increases the area per lipid molecule and decreases the membrane width. With increasing cortisone content, the cortisone molecules start to penetrate the hydrophobic core. At cortisone concentrations of 50 mol% in the simulations (in part d), the membrane’s thickness has decreased such that cortisone molecules in the different leaflets can find partners from the opposite leaflet when they penetrate the hydrophobic core. We propose that these structures are the pre-cursors for the formation of cortisone crystallites. Although the experimental threshold for detecting crystals is 20 mol%, we cannot exclude the possibility that crystals also exist at concentrations below 20 mol% and that the corresponding signals are too small to be detected by diffraction in the present study.

The corresponding structure of a membrane with embedded crystallite is shown in Fig. 4e. The thickness of the hydrophobic core at 50 mol% cortisone of ~25 Å corresponds to about 4 layers of cortisone. The decrease in the lamellar spacing 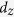 is mainly caused by the decrease in membrane thickness; the small value of 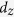 of 48 Å excludes that the cortisone crystallites form outside of the bilayers.

Cortisone is often used to treat inflammation of the joints to function as inflammation reducing agents, which in turn reduces pain in the joints. It is used in the treatment of bursitis, tendonitis, and arthritis in the form of local injections in the knee, shoulder, elbow or back, or systemic injections for inflammation all over the body. Up to 100 mg of cortisone are commonly injected during an anti-inflammatory treatment^35^. From a simple coarse calculation, if we assume that a cell is ~50 *μ*m in diameter, and the average lipid has an area of 65 Å^2^, and a membrane/water partition coefficient of 200, then 100 mg of cortisone could result in up to 10^9^ cells obtaining cortisone/plasma membrane lipid concentrations of ~20 mol%. Therefore, cortisone is likely present in the elevated concentrations studied here. A cortisone flare is a reaction of the body to a cortisone injection, typically 24–48 hours after the injection has been administered. In about 5% of the cases, cortisone is found to crystallize and cause pain around the soft tissue along with the joint lining, in conjunction with strong pain. A flare is typically treated by resting the inflamed area.

In our simulations and experiments, the lipid bilayer was found to serve as a 2-dimensional substrate for cortisone accumulation and crystallization As cortisone is believed to cross the membrane by free diffusion or membrane mediated endocytosis, the steroid can bind to both the inner and outer leaflet of the plasma membrane, thereby initiating crystallization in the manner outlined in Fig. 4 36. As crystals were found to nucleate inside the bilayers, the composition of the membranes, such as cholesterol content and ratio between saturated and unsaturated fatty acids is likely an important parameter. The present study adds to the increasing evidence that drug-membrane interactions are important for modeling drug side-effects.

## Conclusions

Steroid flares, commonly observed after cortisone injection, have been attributed to the formation of microcrystalline corticosteroid. In this study, we show that crystallized cortisone is observed in POPC model membranes at high cortisone concentration. By combining molecular dynamics simulations and X-ray diffraction, we present a potential mechanism for the nucleation of cortisone flares, where the steroid crystals nucleate within the 2-dimensional plane of the lipid membrane.

Cortisone partitions into the head group-tail group interfacial region of the bilayers at low cortisone concentrations, aligning parallel to the membranes. Increasing cortisone concentration results in an increased area per lipid, a decreasing membrane width, and more disordered lipid tails. At concentrations of more than 20 mol% cortisone, the formation of crystalized cortisone inside the hydrophobic membrane core was observed. These crystals form when the membrane thickness has decreased such that cortisone molecules in the different leaflets can find partners from the opposite leaflet, resulting in a non-zero density of cortisone molecules in the bilayer center. The corresponding crystallites orient with the membranes.

## Materials and Methods

### Experimental Details

#### Preparation of Multi-Lamellar Membrane Samples

Highly-oriented multi lamellar membranes were prepared on single-side polished silicon wafers (1 × 1 cm^2^). 1-palmitoyl-2-oleoyl-sn-glycero-3-phosphocholine, (POPC, Avanti Polar Lipids) and Cortisone (17-hydroxy-11-dehydrocorticosterone, Sigma) were mixed at the desired molecular ratio and dissolved in a 1:1 mixture of chloroform (Caledon)/2,2,2-trifluoroethanol (TFE) (Sigma). The final solution concentration was 16 mg/mL.

The wafers were placed in 1,2-dichloromethane (Caledon) within a closed Pyrex dish. Wafers were cleaned by sonication for 30 minutes, which resulted in a hydrophobic silicon surface. The wafers were then removed and rinsed three times thoroughly with alternating methanol and 18.2 MΩ·cm water. The wafers were then dried with pressurized nitrogen gas 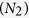 and placed on a tilting incubator set to 310 K. A syringe was used to deposit ~65 *μ*L of the POPC-cortisone solution on the wafer while the tilt (speed 15, tile angle 1°) provided circular flow and even distribution. The samples was allowed to dry for 20 minutes on the tilting incubator.

The samples were then placed in a vacuum for ~24 hours at 298 K to allow for the evaporation of trace solvent. Wafers were stored in a glove box (8% H_2_O) to prevent lipid peroxidation, prior to the X-ray scattering experiments^37^,^38^.

#### X-ray Diffraction Experiment

Out-of-plane and in-plane X-ray scattering data was obtained using the Biological Large Angle Diffraction Experiment (BLADE) in the Laboratory for Membrane and Protein Dynamics at McMaster University. BLADE uses a 9 kW (45 kV, 200 mA) CuK*α* Rigaku Smartlab rotating anode at a wavelength of 1.5418 Å. Both source and detector are mounted on moveable arms such that the membranes stay horizontal during measurements. Focussing, multi layer optics provide a high intensity parallel beam with monochromatic X-ray intensities up to 10^10^ counts/(s × mm^2^). This beam geometry provides optimal illumination of the membrane samples to maximize the scattered signal. By using highly-oriented stacks, the in-plane 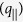 and out-of-plane 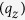 structure of the membranes could be determined independently. Full 2-dimensional reciprocal space maps are shown in Fig. 2. All scans were measured at 301 K and 97% hydration, in the fluid phase of bilayers.

As in-plane features are usually orders of magnitude weaker than the pronounced out-of-plane features, slices 0.03 Å^−1^ < *q*_*z*_ < 0.3 Å^−1^ were integrated to enhance the data quality.

#### Calculation of Electron Densities

The out-of-plane structure of the membrane was determined using specular reflectivity. The relative electron density, *ρ*(z), is approximated by a 1-dimensional Fourier analysis^31^,^39^.


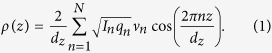


*N* is the highest order of the Bragg peaks observed in the experiment. The integrated peak intensities, 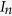, are multiplied by 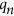 to receive the form factors, 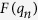
31,39. The bilayer form factor 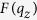, which is in general a complex quantity, is real-valued in the case of centro-symmetry. The phase problem of crystallography, therefore, simplifies to the sign problem 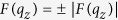 and the phases, 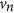, can only take the values ±1. The phases 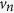 are needed to reconstruct the electron density profile from the scattering data following Equation 1. When the membrane form factor 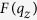 is measured at several 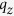 values, a continuous function, 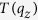, which is proportional to 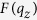, can be fitted to the data^31^,^39^.


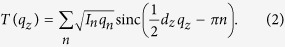


Once an analytical expression for 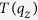 has been determined from fitting the experimental peak intensities, the phases 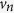 can be assessed from 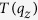. The phase array 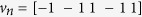 [−1 −1 1 −1 1] was used for all samples. A sample 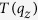 is shown in Fig. 3b.

For POPC and POPC + 5 mol% cortisone, the calculated electron densities, 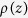, which are initially on an arbitrary scale, were then scaled using the following protocol: The curves were vertically shifted, such that the density at the center of the bilayer was equal to the electron density value determined in the simulations of *ρ*(*z*) = 0.26 e^−^/Å^3^. The curves were then scaled until the total number of electrons within the lipid unit cell across a membrane leaflet, 
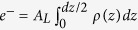
 agrees with the total number of electrons expected based on the sample composition. The area per lipid obtained from simulation were used for the calculation.

The total number of electrons includes contributions from POPC, cortisone, and water molecules. The number of water molecules per lipid was calculated by calculating the approximate volume of water, 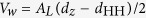, and *ρ*_*w*_ = 0.33 e^−^/Å^3^. For pure POPC and POPC with 5 mol% cortisone, 20 and 21 water molecules per POPC were calculated, respectively. Therefore, for the POPC sample, the integral over the lipid unit cell is assumed to contain one POPC molecule and 20 water molecules. For the 5 mol% cortisone sample, the integral number of electrons over a lipid unit cell in the bilayer is assumed to contain a full lipid molecule, 21 water molecules, and 5% of a cortisone molecule. The procedure follows that outlined by Alsop *et al.*^33^.

### Molecular Dynamics Simulations

#### Simulation setup and parameters

Parameters for POPC were taken from the SLipids force fields^40^, and water was described by the TIP3P model^41^. Cortisone was described by the General Amber Force Field^42^. The cortisone topology was generated by ACPYPE, which builds upon the Antechamber software^43^,^44^. Following the default Antechamber protocol, partial charges were computed on the Hartree-Fock level using the 6-31G* basis set, with the Gaussian 09 software package^45^. The charges were fitted to reproduce the electrostatic potential produced by the quantum chemical calculations with the RESP method^46^. The topology file is available from the author (JSH) upon request.

The lipid membrane simulation systems were generated with the web server MemGen (http://memgen.uni-goettingen.de)^47^. The patches corresponding to cortisone concentrations between 0 and 50% contained between 128 and 100 POPC, between 0 and 100 cortisone, and between 4480 and 6300 water molecules (Supporting Table S1). After energy minimization, each system was simulated for 200 ns. The first 40 ns of all simulations was removed for equilibration. To validate the convergence of the simulations, three independent 200 ns repetitions of the system with 40% cortisone were simulated (Figure S2).

All simulations were carried out using the Gromacs simulation software, version 4.6^48^. During equilibrium simulations, the temperature was controlled at 300 K through velocity rescaling^49^ 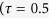 ps), and the pressure was kept at 1 bar using a semi-isotropic weak coupling scheme^50^ 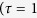 ps). The SETTLE^51^ algorithm was applied to constrain bond lengths and angles of water molecules, and LINCS^52^ was used to constrain all other bond lengths, allowing a time step of 2 fs. Electrostatic interactions were calculated using the particle-mesh Ewald method^53^,^54^, and dispersive interactions were described by a Lennard-Jones potential with a cut-off at 10 Å. To exclude that the cut-off has a major on the results, three additional 200 ns simulations of the system with 40% cortisone were conducted, but simulating instead with a cut-off at 12 Å. The results from these simulations were very similar, suggesting that a 10 Å is acceptable for the present study.

### Umbrella sampling simulations

The simulation protocol was chosen similar to previous studies^55^,^56^. Accordingly, starting structures for the umbrella simulations were taken from randomly chosen snapshots of the last 10 ns of an 20-nanosecond equilibrium simulation of 98 POPC and 5390 water molecules. The membrane normal, *z*, was chosen as reaction coordinate for solute permeation, where 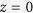 Å is defined by the center of mass (COM) of the lipid molecules. Adjacent umbrella windows were separated by 1 Å, and the umbrella windows spanned the complete space between one bulk water region across the membrane and into the other bulk water region.

Solutes were inserted at the center of the respective umbrella windows. To save computational resources, two or three different umbrella windows were sampled within each simulation, keeping a distance of 35 Å along *z* between cortisone molecules. Water molecules, which overlapped with the solute were removed. Overlaps between the solute and lipid atoms were removed by gradually switching on Lennard-Jones interactions between the solute and the rest of the system within 5000 simulation steps, using soft-core Lennard-Jones potentials and a stochastic dynamics integration scheme. During these insertion simulations only, a large virtual site atom was added to the center of every aromatic ring. That procedure ensured that lipid tails were fully repelled from the aromatic rings of the solutes. Subsequently, the energy of each structure was minimized.

A harmonic umbrella potential acting on the center of mass of the solute was applied (force constant 2000 kJ mol^−1^nm^−2^). Each umbrella simulation was carried out for 80 ns. The temperature was set to 300 K through a stochastic dynamics integrator 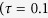 ps). The pressure was controlled at 1 bar using a semi-isotropic weak coupling scheme^50^, scaling the box in the *x*-*y* plane only, but keeping the box dimension in *z*-direction fixed. After removing the first 10 ns for equilibration, the PMFs were computed with the weighted histogram analysis method (WHAM)^57^, as implemented in the g_wham software^58^. First, non-periodic and non-symmetrized PMFs were computed. These PMFs were reasonably symmetric with respect to the membrane center and exhibited only a small offset between the two bulk water regimes, suggesting that the PMFs were converged. Subsequently, a periodic PMF was computed and symmetrized with respect to the membrane center 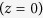. Because the PMF 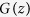 was defined to zero in bulk water, the membrane/water partition coefficient could be computed via 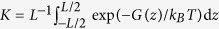, where 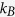 denotes the Boltzmann constant and *T* the temperature.

## Additional Information

**How to cite this article**: Alsop, R. J. *et al.* The Lipid Bilayer Provides a Site for Cortisone Crystallization at High Cortisone Concentrations. *Sci. Rep.* **6**, 22425; doi: 10.1038/srep22425 (2016).

## Supplementary Material

Supplementary Information
