# Supplementary material for: The Lipid Bilayer Provides a Site for Cortisone Crystallization at High Cortisone Concentrations
Source: Sci Rep. 2016 Mar 3;6:22425. doi: 10.1038/srep22425 (PMC4776104; doi:10.1038/srep22425)
Supplement: Supplementary Information [file srep22425-s1.pdf]

The Lipid Bilayer Provides a Site for Cortisone Crystallization at High Cortisone Concentrations

Richard J. Alsop,[1] Adree Khondker,[1] Jochen S. Hub,[2,*] and Maikel C. Rheinstädter[1,†]

[1] *Department of Physics and Astronomy,*
McMaster University, Hamilton, Ontario, Canada
[2] *Georg-August-University Göttingen,*
Institute for Microbiology and Genetics, Göttingen, Germany

[*] jhub@gwdg.de; Institute for Microbiology and Genetics, Georg-August-University Göttingen, Justus-von-Liebig-Weg 11, 37077 Göttingen, Germany; Phone: +49-(0)551-39-14189

[†] rheinstadter@mcmaster.ca; Department of Physics and Astronomy, McMaster University, ABB-241, 1280 Main Street West, Hamilton, Ontario L8S 4M1, Canada; Phone: +1-(905)-525-9140-23134, Fax: +1-(905)-546-1252

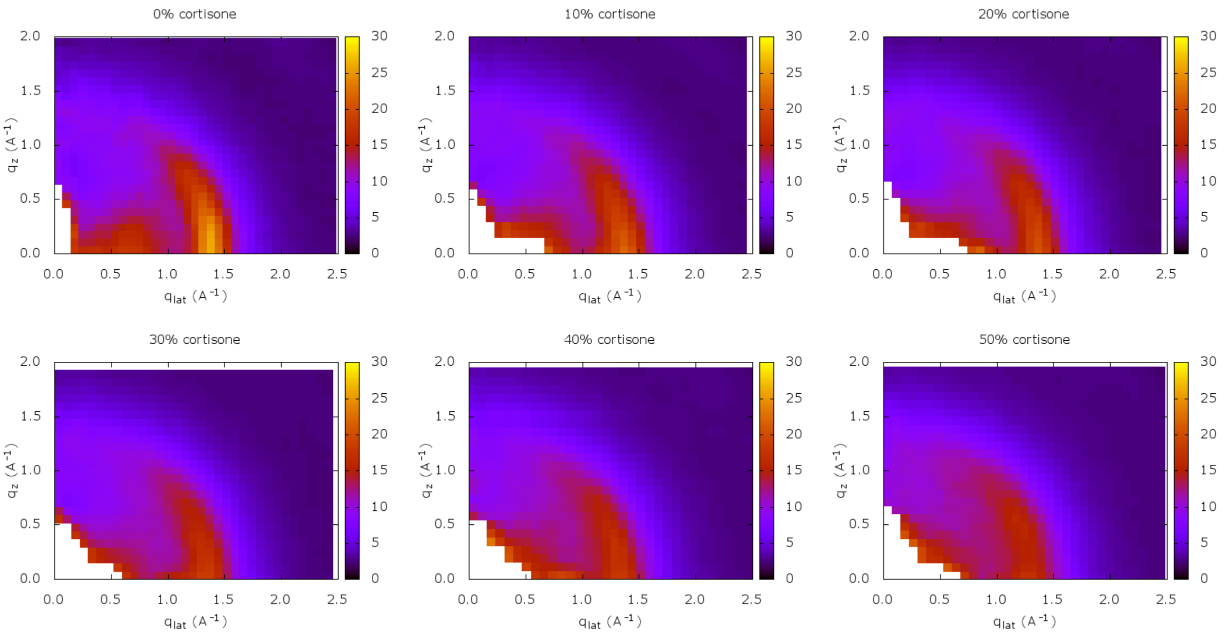

FIG. S1. Two-dimensional X-ray intensities $S(q_{\text{lat}}, q_z)$ computed from molecular dynamics simulations, at cortisone concentrations between 0 and 50% (see figure titles). $S(q_{\text{lat}}, q_z)$ was computed from POPC and cortisone atoms. $S(q_{\text{lat}}, q_z)$ is plotted as a function of momentum transfer q_{lat} in the membrane plane and q_z perpendicular to the membrane plane. In agreement with the experimental data, the chain correlation peak at $q = \sqrt{q_{\text{lat}}^2 + q_z^2} \approx 1.4\,\text{Å}^{-1}$ is reduced and smeared out upon increasing cortisone content. In contrast to the experimental data, no additional peaks due to cortisone crystals appear because crystals did not form within the accessible simulation time.

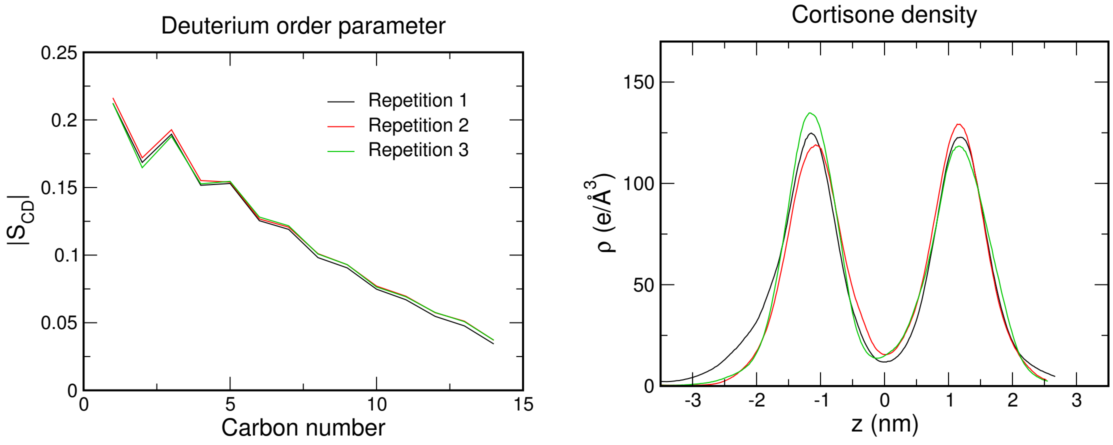

FIG. S2. Deuterium order parameter (left) and cortisone density (right) computed from three independent 200 ns simulations of the simulation system with 40% cortisone (after removing the first 40 ns for equilibration). The agreement suggests that the conformational sampling accessible on this time scale is converged. However, the analysis does not exclude the possibility of the formation of cortisone crystals on longer time scales.

TABLE 1. Summary of equilibrium MD simulations: approximate cortisone content, number of POPC N_L, coritsone N_c, and water molecules N_w, simulation time, number of repetitions.

cortisone content	N_L / N_c / N_w	t_{sim} (ns)	repetitions
0%	128 / 0 / 4480	100	1
5%	132 / 8 / 4900	200	1
10%	122 / 14 / 4760	200	1
20%	120 / 30 / 5250	200	1
30%	126 / 54 / 6300	200	1
40%	108 / 72 / 5400	200	3
50%	100 / 100 / 6000	200	1
